# Suicidal Ideation Is Associated with Altered Variability of Fingertip Photo-Plethysmogram Signal in Depressed Patients

**DOI:** 10.3389/fphys.2017.00501

**Published:** 2017-07-19

**Authors:** Ahsan H. Khandoker, Veena Luthra, Yousef Abouallaban, Simanto Saha, Khawza I. U. Ahmed, Raqibul Mostafa, Nayeefa Chowdhury, Herbert F. Jelinek

**Affiliations:** ^1^Department of Biomedical Engineering, Khalifa University Abu Dhabi, United Arab Emirates; ^2^Department of Electrical and Electronic Engineering, University of Melbourne VIC, Australia; ^3^American Center for Psychiatry and Neurology Abu Dhabi, United Arab Emirates; ^4^Department of Electrical and Electronic Engineering, United International University Dhaka, Bangladesh; ^5^School of Community Health and Centre for Research in Complex Systems, Charles Sturt University Albury, NSW, Australia; ^6^Australian School of Advanced Medicine, Macquarie University Sydney, NSW, Australia

**Keywords:** major depressive disorder, suicidal ideation, photo-plethysmogram, pulse wave, tone-entropy analysis, pulse wave variability

## Abstract

Physiological and psychological underpinnings of suicidal behavior remain ill-defined and lessen timely diagnostic identification of this subgroup of patients. Arterial stiffness is associated with autonomic dysregulation and may be linked to major depressive disorder (MDD). The aim of this study was to investigate the association between arterial stiffness by photo-plethysmogram (PPG) in MDD with and without suicidal ideation (SI) by applying multiscale tone entropy (T-E) variability analysis. Sixty-one 10-min PPG recordings were analyzed from 29 control, 16 MDD patients with (MDDSI+) and 16 patients without SI (MDDSI−). MDD was based on a psychiatric evaluation and the Mini-International Neuropsychiatric Interview (MINI). Severity of depression was assessed using the Hamilton Depression Rating Scale (HAM-D). PPG features included peak (systole), trough (diastole), pulse wave amplitude (PWA), pulse transit time (PTT) and pulse wave velocity (PWV). Tone (Diastole) at all lags and Tone (PWA) at lags 8, 9, and 10 were found to be significantly different between the MDDSI+ and MDDSI− group. However, Tone (PWA) at all lags and Tone (PTT) at scales 3–10 were also significantly different between the MDDSI+ and CONT group. In contrast, Entropy (Systole), Entropy (Diastole) and Tone (Diastole) were significantly different between MDDSI− and CONT groups. The suicidal score was also positively correlated (*r* = 0.39 ~ 0.47; *p* < 0.05) with systolic and diastolic entropy values at lags 2–10. Multivariate logistic regression analysis and leave-one-out cross-validation were performed to study the effectiveness of multi-lag T–E features in predicting SI risk. The accuracy of predicting SI was 93.33% in classifying MDDSI+ and MDDSI− with diastolic T-E and lag between 2 and 10. After including anthropometric variables (Age, body mass index, and Waist Circumference), that accuracy increased to 96.67% for MDDSI+/− classification. Our findings suggest that tone-entropy based PPG variability can be used as an additional accurate diagnostic tool for patients with depression to identify SI.

## Introduction

Over 800,000 people die due to suicide every year and there are many more who attempt suicide (WHO, 2016). The global suicide rate was highest in low- and middle-income countries in 2015 with 78% and suicide accounted for 1.4% of all deaths worldwide, making it the 17th leading cause of death in 2015 (WHO, 2016). Over 90% of people who die by suicide have clinical depression or another diagnosable mental disorder (Leenaars, 1996; Soloff et al., 2000). Depressed individuals with suicidal risk might have been undiagnosed because of persistent ignorance about depression. Misperceptions by the public, and even some health providers, who interpret the disease to be a personal weakness in character, often lead to painful stigmatization of sufferers, and treatment avoidance. In the United Arab Emirates, apathy toward seeking professional mental health services by UAE citizens is associated with societal stigma, lack of awareness of mental health and lack of confidence in mental health-care providers (Chowdhury, 2016). Therefore, it has been recommended that physiological evidence-based diagnostic methods and interventions need to be implemented at a population, sub-population and individual level to prevent suicide and suicide attempts. Suggestions have been made that alternative diagnostic tools be used in conjunction with a psychiatric evaluation, or as a stand-alone screening, as part of a community health initiative; which may provide an additional source of information, allowing individuals at risk to be assessed.

Pathophysiological factors such as increased arterial stiffness adversely affect cognitive neurological function, with high pulsatile blood flow damaging cerebral micro-vessels. Progressive endothelial dysfunction, edema, hemorrhage and inflammation may then lead to depressive symptomatology as part of the clinical spectrum (Henry-Feugeas, 2009). Previous research has shown that patients with suicidal tendencies exhibited significant and more pronounced cardiac vagal withdrawal compared to both the patients without suicidal ideation and the healthy control group (Chang et al., 2012). Another study has suggested that suicidal ideation is related to altered serotonergic function, which also influences vascular tone and is possibly influenced by vagal output (Wells, 2008).

Arterial stiffness measures the rigidity of the arterial wall. Several different methods of assessing arterial stiffness have been proposed in the past (Mackenzie et al., 2002), including pulse pressure (difference between systolic and diastolic blood pressures), pulse wave velocity (PWV, which is speed at which the forward pressure wave is transmitted from the aorta through the vascular tree), vascular distensibility and compliance of brachial, femoral and carotid arteries by Ultrasound and MRI derived indices such as the stiffness index (β) [i.e., ratio of ln(systolic/diastolic pressures) to (relative change in diameter)], augmentation index (the difference between the second and first systolic peaks) from arterial pressure waveform obtained by tonometry (Sato et al., 1993).

Photoplethysmography (PPG) has been used to record the finger peripheral blood volume pulse. This technique resembles that of pulse oximetry, and measures the transmission of infrared light through the finger and detecting changes in blood flow, which is recorded as a blood volume waveform. It has been used to develop a stiffness index and a reflection index that are thought to reflect systemic arterial stiffness (Chowienczyk et al., 1999). A previous study (Bortolotto et al., 2000) that compared PPG of the finger blood volume pulse with pulse wave velocity (PWV) (Asmar et al., 1999), found that PWV correlated more closely with the expected influences of age and atherosclerosis, which influence vascular compliance. PPG provides potential uses in a psychiatric, mental health setting as it has the advantage of being relatively simple to administer and equipment is easily portable.

We hypothesize that suicidal ideation in depression may be associated with arterial stiffness as measured by PPG wave parameters (Systole, Diastole and Pulse Wave Amplitude, Pulse Transit Time, Pulse Wave Velocity) extracted from fingertip PPG signals. Recently, Khandoker et al. (2010) used tone-entropy analysis of beat-to-beat timing (RR intervals) in a study of autonomic control of heart rate variability (HRV) and demonstrated that sympatho-vagal balance can be detected in the time domain by tone-entropy (T-E). Tone reflected the sympatho-vagal balance of heart rate and changed consistently in post-exercise recovery as the (inhibiting) parasympathetic division became predominant (Oida et al., 1997). As with heart rate control, it was hypothesized here that T-E analysis would prove useful in revealing beat-to-beat pulse wave variability that is masked using traditional linear methods. T-E analysis has the advantage that it is not influenced by either the time period of data acquisition or baseline wander of the signal. An extension of T-E algorithm is multi-lag T-E analysis, which provides a more accurate characterization of the time signal as the heart beat influences beats 6-10 beats downstream, reflecting parasympathetic (up to 4 beats) and sympathetic influences (up to 10 beats) and hence also the pulse wave of interest in this study (Claudia et al., 2003; Karmakar et al., 2013).

Therefore, the aim of this study was to investigate the association of arterial pulse wave parameters in clinically depressed patient with and without suicidal ideation and a control group by using a multi-lag tone entropy analysis of PPG recordings.

## Methods

### Subjects and psychiatric assessment

Thirty-two unmedicated MDD patients participated in the research. Sixteen were Suicidal Ideation (SI) positive [MDDSI+] and 16 MDD patients were without SI [MDDSI−]. Twenty-nine non-depressed participants were also included in the study at the American Center for Psychiatry and Neurology in Abu Dhabi. Al Ain District Ethics Committee approved the study (14/28) and all participants gave written consent. Diagnoses of the severity of MDD were made by the consultant psychiatrist (VL) using the Mini-International Neuropsychiatric Interview (MINI) version 5 (Sheehan et al., 1998); and the severity of clinical depression was assessed using the Hamilton Depression Rating Scale (HAM-D) (Williams, 1988). Exclusion criteria included: inadequate reading or verbal fluency in Arabic or English; significant cognitive impairment; other primary psychiatric diagnosis; active medical diagnoses of ischemic heart disease; diabetes; or the presence of inflammatory illness within the preceding two years. All patients recruited in this study were diagnosed with MDDSI+/− at their first visit to the psychiatric clinic by the psychiatrist. The control group was interviewed by the psychiatrist for screening of mental illnesses, and deciding whether further psychiatric tests were required to confirm the diagnoses. In this study, none of the participants in the healthy control group were identified with mental health issues, and therefore did not complete the MINI interview and the other questionnaires. Table 1 summarizes the demographics of participants for the three group cohorts.

**Table 1:** Patient's demographics and psychiatric scores.

**Variable**	**MDDSI+**	**MDDSI−**	**CONT**	***p*-value**
**N**	**16**	**16**	**29**	
Gender male,%	5 (31.25%)	3 (18.75%)	12 (41.37%)	
Age (years)	37.50 ± 10.37	32.31 ± 6.95	28.00 ± 6.35	0.00^#^
WC (cm)	95.50 ± 16.64	82.73 ± 10.89	76.24 ± 9.61	0.00^*^^#^
Height (cm)	164.60 ± 10.22	164.44 ± 8.41	162.28 ± 9.78	0.66
BMI (kg/m^2^)	34.77 ± 25.65	24.98 ± 3.27	23.89 ± 3.44	0.03^#^
SBP (mmHg)	114.00 ± 14.04	112.50 ± 13.90	110.34 ± 8.65	0.60
DBP (mmHg)	72.67 ± 7.99	71.25 ± 7.19	71.72 ± 7.59	0.87
BDI	36.06 ± 12.23	33.19 ± 11.55		0.50
GAD7	17.94 ± 11.03	14.56 ± 4.21		0.26
PHQ9	21.06 ± 12.49	18.81 ± 4.48		0.50
Suicidal score	17.35 ± 7.25	0.92 ± 1.75		0.00^*^

* MDDSI+ and MDDSI−;

# *CONT and MDDSI+. BMI, Body Mass Index; SBP, Systolic Blood Pressure; DBP, Diastolic Blood Pressure; BDI, Beck Depression Inventory; GAD7, General Anxiety Disorder; PHQ, Patients Health Questionnaire*.

The MINI is a well-validated, standardized, and structured short diagnostic interview that provides an evaluation of psychiatric diagnoses according to the American Psychiatric Association's Diagnostic and Statistical Manual for Mental Disorders; (Fifth edition); text revision (DSM-V); and ICD-10 criteria (Sheehan et al., 1998). Furthermore, the C module of the MINI, was used to estimate suicidal ideation among participants. This module is made up of nine questions. Questions C1–C8 explore and assess events in the past month, while question C9 takes account of suicide attempts made in a patient's lifetime. The module assesses suicidal behaviors including intent, planning and actual attempts at suicide. The minimum score for the C module is zero (0) and the maximum score is 38. Scores ranging from 1 to 8 points indicate a low suicide risk, whereas 9–16 points indicate moderate suicidal risk, and scores greater than 17 points indicate high suicide risk. Higher scores indicate greater suicidal ideation. In this study, MDD patients with scores more than 9 were considered to be MDDSI+ patients. All participants except the control group completed the Depression, Anxiety and Stress Scales (PHQ-9, GAD 7, and BDI) (Beck et al., 1988), which are reliable and valid self-report measures of depression, anxiety, and stress severity (Table 1).

### Experiments

All participants underwent a supine resting ECG, respiration recording, and finger PPG (Powerlab ADInstruments). Physiological signals were recorded over 10 min and the ECG recorded using a lead II configuration (Powerlab, AdInstruments, Australia). Data were captured on Labchart 7.1 with a sampling rate set at 1,000 Hz and a notch filter at 50 Hz. Temperature and humidity of the room were set at 24°C and 55% respectively. Participants were asked to abstain from coffee and cigarettes on the day of testing. However, physical activity and types of food intake were not controlled in this study, due to the nature of the mental health status of the patients to avoid anxiety and stress.

### Feature extraction from arterial pulse signals

**Figure 1:**
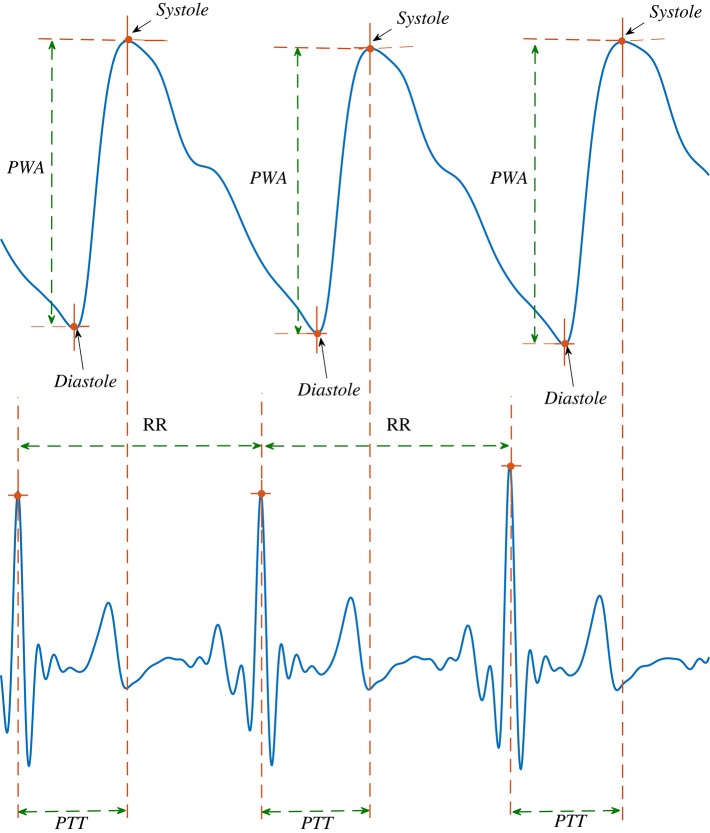
Feature (Systole, Diastole, PWA and PTT) extraction from simultaneously recorded ECG and PPG signals.

A typical example of a PPG signal with different features that can be extracted for analysis is shown in Figure 1.

#### Systole and diastole

The peak and trough of each cycle of the arterial pulse signal represent the systole and diastole phase of the cardiac cycle respectively. The vertical distance between systole and diastole points is measured as pulse wave amplitude (PWA). The pulse transit time (PTT) is calculated as the time difference between the R peaks of the ECG signal and consecutive systole points (Figure 1). First all systoles and diastoles are detected as the local maximum and minimum points of the pulse signal. Then PWA is calculated by subtracting diastoles from immediately preceding systoles.

The Pulse Wave Velocity (PWV) was calculated using the following formula (Davies and Struthers, 2003): PWV (cm/ms) = [height (cm) × BDC]/PTT (ms)]. BDC = body correlation factor, and height = body length. BDC is 0.5 for adults when recorded at the finger for detection of the peripheral pulse wave as used in this study.

### Multi-lag T-E analysis of features extracted from PPG signal

Let, X_S_ represents the series corresponding to any of the Systole, Diastole, PWA, PTT, and PWV and defined as follows

(1) $${\text{X}}_{\text{S}}=\{{\text{X}}_{1,}{\text{X}}_{2},......,{\text{X}}_{\text{N}}\}$$

where, S = Systole/Diastole/PWA/PTT/PWV, i = corresponding value in the ith cardiac cycle (beat) and N = the number of beats used in the analysis (N = 400).

In TE analysis, the percentile changes as beat to beat variation in Systole, Diastole, PWA, PTT or PWV are calculated after normalization by the previous beat as follows

(2) $$\;{\text{PI}}_{\text{i}}\;=\;\frac{{\text{X}}_{\text{i}}-{\text{X}}_{\text{i}+1}}{{\text{X}}_{\text{i}}}\times 100\;$$

The beat to beat escalation, i.e., X_i+1_ > X_i_ refers to the increased Systole and Diastole individually whereas PWA depicts the increased difference between Systole and Diastole. In such a case, higher PTT depicts higher traveling time for blood between two arterial sites. In contrast, higher PWV depicts lesser traveling time for blood between two arterial sites.

Tone is defined as a first order moment (arithmetic average) of the PI time series as

(3) $$\text{Tone }=\;\frac{1}{\text{N}-1}\sum_{\text{i}=1}^{\text{N}-1}{\text{PI}}_{\text{i}}\;$$

**Figure 2:**
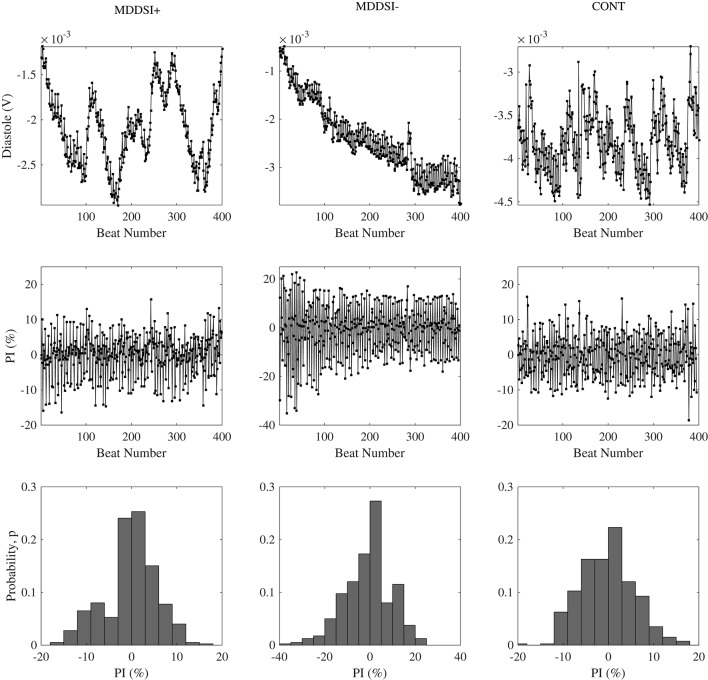
Typical Diastole series **(Top)**, PI time series deduced from above **(Middle)**, and its probability distributions in histogram **(Bottom)** selected in each group (MDDSI+, MDDSI−, and CONT).

Entropy is defined from the probability distribution of the PI series by using Shannon's formula

(4) $$\text{Entropy=}-\sum_{\text{i}=1}^{\text{n}}\text{p}\left(\text{i}\right){\text{log}}_{2}\text{p}\left(\text{i}\right)\;$$

where p(i) is the probability distribution of PI having values in the range i < PI < i+1, where i is an integer and n is the number of bins and p(i) ≠ 0. Figure 2 demonstrates examples of diastolic time series, its PI series and the probability distribution of PI series for selected subjects from MDDSI+, MDDSI− and CONT groups.

**Figure 3:**
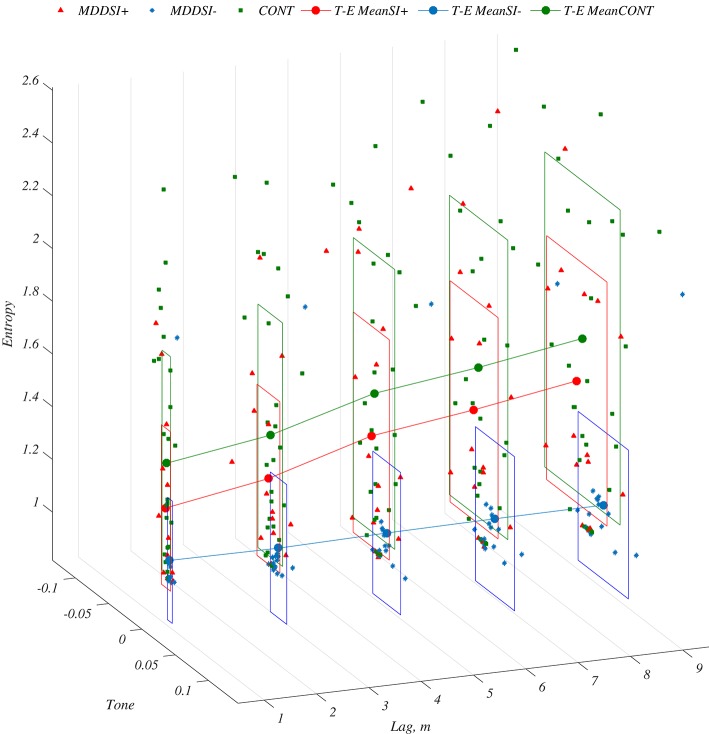
Visualization of multi-lag tone and entropy (T-E) features extracted from Diastole series at 3D plot corresponding to MDDSI+ (Δ), MDDSI− (■), and CONT (•) groups respectively, along with rectangles representing Mean ± SD.

For multi-lag T-E analysis, we have introduced the *lag* (*m*) in Equation (2). In the multi-lag T-E analysis, PI is expressed as the percentile change of the ith and (i+m)th beats values with respect to the ith beat value and is defined as:

(5) $${\text{PI}}_{\text{i}}\;=\;\frac{{\text{X}}_{\text{i}}\;-\;{\text{X}}_{\text{i}+\text{m}}}{{\text{X}}_{\text{i}}}\times 100\;$$

where, m is an integer and m = 1 represents the conventional T-E analysis. The detailed methodology of conventional and multi-lag T-E analysis has been described in previous reports (Khandoker et al., 2010; Karmakar et al., 2013). An important benefit of conventional (*lag, m* = *1*) T-E analysis is that it is not influenced by the time period of data acquisition and increasing lag time reintroduces the sensitivity to recording length. We analyzed the variation of tone and entropy values with a range of lags (1 ≤ m ≤ 10).

Several time domain features were also included in the analysis: mean, standard deviation (SD) and the square root of the mean squared difference (RMSSD) (Task force of the European Society of Cardiology, 1996) of the successive beat values of all PPG features.

### Statistics

A Kruskal-Wallis nonparametric test of covariance (Quade test) (Quade, 1967; Maxwell et al., 1993) was performed to check the differences among healthy (CONT), MDD patients with suicidal ideation (MDDSI+), and MDD patients without suicidal ideation (MDDSI−) after adjusting the models for age, body mass index (BMI) and waist circumference (WC) as covariates. Values of *p* < 0.05 were considered as significant. Bonferroni correction for multiple pairwise testing was used after p value was significant (*p* < 0.05). Correction was set at (*p* < 0.0166), calculated by dividing 0.05 by three groups. A non-parametric test was used because of the non-Gaussian distribution of the variables based on the Lilliefors test. The relative importance of T-E features was determined by receiver-operating curve (ROC) (Hanley and McNeil, 1983), with the area under the curve for each feature represented by the ROC area. A ROC area value of 0.5 indicates that the distributions of the variables are similar in both populations. Conversely, a ROC area value of 1.0 would mean that the distributions of the variables of the two populations do not overlap at all. Based on the ROC analysis, linear classifiers were then built for classifying each of the three groups in pairwise manner, i.e., MDDSI+ vs. MDDSI-, MDDSI- vs. CONT and CONT vs. MDDSI+. The classifiers performances were measured using the parameters like Optimal Threshold, Sensitivity, Specificity and Accuracy in addition to the ROC area (Fawcett, 2004).

### Multivariate logistic regression analysis

In order to predict MDD patients with suicidal risk from the PPG parameters, multivariate logistic linear models were computed using the generalized linear model (GLM) function from MATLAB software. A stepwise regression analysis was employed based on individual and all possible combinations of parameters including Tone-Entropy with/without anthropometric (Age, BMI, and Waist Circumference, WC) features. The three categorical models [Model 1: MDDSI+/MDDSI−, Model 2: MDDSI−/CONT, and Model 3: MDDSI+/CONT] and one continuous model (taking suicidal scores of 30 MDDSI+/− patients), which contained an intercept and linear terms of estimated features were also generated with and without including anthropometric features. Stepwise regression automatically adds to or removes from the model in a forward and backward process non-significant parameters to determine a final model. The criterion applied was the *p*-value for an *F*-test that indicates the change in the sum of squared error by adding or removing the parameters. Root mean squared error, R-squared, adjusted R-squared and the *F*-test statistic results vs. a constant model were calculated for the regression of each set of parameters. By leaving one case out for regression analysis and then applying the model to that case, area under the ROC curve (AUC) accuracy, positive predictive value (PPV) and negative predictive value (NPV) were also computed for all three models. The models were designed and presented in **Table 8** with an intercept and estimated coefficients with a *p*-value less than or equal to 0.05 considered statistically significant. The selection of the best model relied on the Akaike information criterion (AIC). The correlation coefficient was used to measure the performance of continuous model based estimation of suicidal scores.

## Results

Table 1 summarizes the clinical variables for all the patients. WC values were significantly higher in the MDDSI+ group than in the MDDSI- group. Age and BMI of the MDDSI− group were significantly higher compared to those in the CONT group. Table 2 shows that mean, SD and RMSSD of Systole, Diastole and PWA values of the MDDSI− group were significantly lower compared to the CONT group (*p* < 0.05). As for PTT and PWV features, SD and RMSSD were found to be significantly higher in the MDDSI+ compared to the CONT group.

**Table 2:** Mean ± SD values of time domain features.

**Feature**		**MDDSI+**	**MDDSI−**	**CONT**	**Sig**.	***p*-value**
		**Mean ± SD**	**Mean ± SD**	**Mean ± SD**		
Systole (volt × E-04)	Mean	45.8 ± 40.8	16.7 ± 14.1	34.8 ± 30.4	^∧^	0.02
	SD	8.22 ± 6.48	4.5 ± 3.68	8.42 ± 6.91	^∧^	0.01
	RMSSD	3.31 ± 2.06	2.11 ± 1.63	4.72 ± 2.64	^∧^#	1.22E-04
Diastole (volt × E-04)	Mean	−39.8 ± 30.8	−16.6 ± 14.5	−33.5 ± 24.6	^∧^	0.01
	SD	7.24 ± 5.16	4.46 ± 4.15	7.33 ± 5.30	^∧^	0.01
	RMSSD	3.62 ± 2.27	2.09 ± 1.64	4.62 ± 2.71	^∧^#	3.72E-04
PWA (volt × E-04)	Mean	85.6 ± 71.0	33.3 ± 28.3	68.4 ± 54.8	^∧^#	0.01
	SD	14.8 ± 11.6	8.66 ± 7.67	14.6 ± 11.9	^∧^	0.01
	RMSSD	5.12 ± 3.64	3.25 ± 2.51	7.19 ± 4.44	^∧^#	3.49E-04
PTT (ms)	Mean	361 ± 41.9	381 ± 45.7	364 ± 42.2	#	2.94E-04
	SD	15.3 ± 10.7	20.6 ± 14.4	24.6 ± 16.1	#	0.01
	RMSSD	13.0 ± 13.2	19.4 ± 15.1	27.3 ± 20.4	#	0.01
PWV (cm/ms) × E-03	Mean	232 ± 30.5	220 ± 36.8	227 ± 30.5		0.14
	SD	9.51 ± 6.06	12.3 ± 8.24	14.4 ± 9.12	#	0.01
	RMSSD	7.93 ± 7.67	11.3 ± 8.38	15.8 ± 11.4	#	0.01

^*^Significantly different between MDDSI+ and MDDSI−;

∧ significantly different between MDDSI− vs. CONT;

# *significantly different between CONT and MDDSI+*.

Tables 3, 4 summarizes group comparisons for Tone and Entropy results. For clinical review the most important diagnostic is risk of identifying suicidal ideation in patients with MDD. Tone diastole performed best for lags 1–3, whereas Tone PWA for longer lags of 8–10 was also significant when comparing MDDSI− to MDDSI+. None of the Entropy results were significant for differentiating between MDDSI− to MDDS+ but did show significant differences between MDDSI− and CONT as well as MDDSI+ and CONT.

**Table 3:** Mean ± SD values of multilag tones.

**Feature**	**Lag, m**	**MDDSI+**	**MDDSI−**	**CONT**	**Sig**.	***p*-value**
		**Mean ± SD**	**Mean ± SD**	**Mean ± SD**		
Tone (Systole)	1	−0.002 ± 0.005	−0.004 ± 0.004	−0.001 ± 0.006		0.23
	2	−0.001 ± 0.010	−0.006 ± 0.008	−0.002 ± 0.012		0.15
	3	−0.001 ± 0.014	−0.009 ± 0.011	−0.002 ± 0.019		0.11
	4	−0.001 ± 0.019	−0.012 ± 0.014	−0.003 ± 0.026		0.08
	5	−0.001 ± 0.023	−0.014 ± 0.018	−0.003 ± 0.032		0.06
	6	−0.002 ± 0.027	−0.017 ± 0.021	−0.004 ± 0.038		0.06
	7	−0.002 ± 0.031	−0.020 ± 0.025	−0.004 ± 0.044		0.06
	8	−0.003 ± 0.036	−0.023 ± 0.028	−0.004 ± 0.050		0.06
	9	−0.003 ± 0.040	−0.026 ± 0.031	−0.005 ± 0.056		0.06
	10	−0.004 ± 0.044	−0.028 ± 0.034	−0.005 ± 0.062		0.05
Tone (Diastole)	1	−0.003 ± 0.007	0.003 ± 0.004	−0.003 ± 0.006	^*^^∧^	1.50E-03
	2	−0.005 ± 0.012	0.006 ± 0.008	−0.003 ± 0.012	^*^^∧^	1.55E-03
	3	−0.007 ± 0.017	0.009 ± 0.012	−0.004 ± 0.018	^*^^∧^	3.41E-03
	4	−0.009 ± 0.023	0.011 ± 0.016	−0.004 ± 0.024	^*^^∧^	0.01
	5	−0.009 ± 0.027	0.014 ± 0.021	−0.005 ± 0.031	^*^^∧^	0.01
	6	−0.010 ± 0.031	0.017 ± 0.025	−0.005 ± 0.038	^*^^∧^	0.01
	7	−0.012 ± 0.036	0.019 ± 0.029	−0.005 ± 0.044	^*^^∧^	0.01
	8	−0.013 ± 0.041	0.022 ± 0.034	−0.006 ± 0.050	^*^^∧^	0.01
	9	−0.015 ± 0.045	0.025 ± 0.038	−0.006 ± 0.057	^*^^∧^	0.02
	10	−0.016 ± 0.050	0.027 ± 0.042	−0.006 ± 0.062	^*^	0.02
Tone (PWA)	1	−0.563 ± 0.763	−1.186 ± 1.276	−1.156 ± 1.150	#	0.06
	2	−0.710 ± 0.849	−1.523 ± 1.329	−1.506 ± 1.598	#	0.05
	3	−0.752 ± 0.919	−1.701 ± 1.278	−1.596 ± 1.382	#	0.03
	4	−0.864 ± 1.061	−2.039 ± 1.434	−1.747 ± 1.407	#	0.02
	5	−0.985 ± 1.147	−2.335 ± 1.514	−2.025 ± 1.688	#	0.02
	6	−1.091 ± 1.293	−2.668 ± 1.681	−2.182 ± 1.773	#	0.01
	7	−1.175 ± 1.372	−3.012 ± 1.792	−2.397 ± 1.979	#	0.01
	8	−1.290 ± 1.505	−3.352 ± 1.989	−2.621 ± 2.283	^*^#	0.01
	9	−1.399 ± 1.640	−3.650 ± 2.187	−2.736 ± 2.448	^*^#	0.01
	10	−1.500 ± 1.746	−4.000 ± 2.413	−2.909 ± 2.686	^*^#	0.01
Tone (PTT)	1	−0.116 ± 0.189	−0.212 ± 0.250	−0.409 ± 0.502		0.09
	2	−0.142 ± 0.226	−0.223 ± 0.237	−0.471 ± 0.555		0.06
	3	−0.146 ± 0.237	−0.245 ± 0.253	−0.458 ± 0.521	#	0.05
	4	−0.148 ± 0.243	−0.278 ± 0.292	−0.472 ± 0.522	#	0.03
	5	−0.163 ± 0.269	−0.300 ± 0.320	−0.507 ± 0.572	#	0.04
	6	−0.164 ± 0.281	−0.309 ± 0.331	−0.521 ± 0.576	#	0.03
	7	−0.162 ± 0.279	−0.324 ± 0.363	−0.534 ± 0.613	#	0.03
	8	−0.164 ± 0.275	−0.344 ± 0.387	−0.550 ± 0.628	#	0.02
	9	−0.179 ± 0.306	−0.334 ± 0.396	−0.546 ± 0.625	#	0.04
	10	−0.191 ± 0.330	−0.328 ± 0.384	−0.551 ± 0.636	#	0.05
Tone (PWV)	1	−0.122 ± 0.188	−0.192 ± 0.261	−0.403 ± 0.526		0.14
	2	−0.142 ± 0.205	−0.203 ± 0.280	−0.478 ± 0.597		0.11
	3	−0.152 ± 0.215	−0.191 ± 0.270	−0.459 ± 0.557		0.12
	4	−0.155 ± 0.224	−0.209 ± 0.343	−0.470 ± 0.574		0.1
	5	−0.163 ± 0.230	−0.215 ± 0.349	−0.492 ± 0.620		0.13
	6	−0.162 ± 0.225	−0.227 ± 0.379	−0.507 ± 0.628		0.1
	7	−0.159 ± 0.215	−0.232 ± 0.440	−0.513 ± 0.644		0.07
	8	−0.161 ± 0.212	−0.248 ± 0.446	−0.517 ± 0.646		0.06
	9	−0.171 ± 0.218	−0.248 ± 0.463	−0.504 ± 0.612		0.07
	10	−0.175 ± 0.220	−0.252 ± 0.488	−0.491 ± 0.604		0.09

* Significantly different between MDDSI+ and MDDSI−;

∧ Significantly different between MDDSI− vs. CONT;

# *Significantly different between CONT and MDDSI+*.

**Table 4:** Mean ± SD values of multilag entropy.

**Feature**	**Lag, m**	**MDDSI+**	**MDDSI−**	**CONT**	**Sig**.	***p*-value**
		**Mean ± SD**	**Mean ± SD**	**Mean ± SD**		
Entropy (Systole)	1	1.213 ± 0.258	1.080 ± 0.176	1.342 ± 0.307	^∧^#	4.38E-05
	2	1.279 ± 0.282	1.086 ± 0.211	1.407 ± 0.352	^∧^#	3.53E-05
	3	1.301 ± 0.290	1.082 ± 0.230	1.457 ± 0.391	^∧^#	2.30E-05
	4	1.341 ± 0.329	1.091 ± 0.232	1.516 ± 0.443	^∧^#	3.63E-05
	5	1.388 ± 0.367	1.105 ± 0.238	1.557 ± 0.480	^∧^#	3.23E-05
	6	1.439 ± 0.409	1.103 ± 0.234	1.593 ± 0.509	^∧^#	2.77E-05
	7	1.456 ± 0.427	1.119 ± 0.231	1.634 ± 0.542	^∧^#	2.93E-05
	8	1.473 ± 0.453	1.118 ± 0.235	1.666 ± 0.561	^∧^#	2.78E-05
	9	1.502 ± 0.468	1.125 ± 0.227	1.676 ± 0.579	^∧^#	2.99E-05
	10	1.533 ± 0.479	1.130 ± 0.240	1.703 ± 0.599	^∧^#	2.79E-05
Entropy (Diastole)	1	1.259 ± 0.302	1.072 ± 0.232	1.429 ± 0.417	^∧^#	1.53E-04
	2	1.324 ± 0.344	1.092 ± 0.255	1.472 ± 0.460	^∧^#	2.45E-04
	3	1.317 ± 0.325	1.082 ± 0.265	1.486 ± 0.462	^∧^#	8.28E-05
	4	1.364 ± 0.365	1.089 ± 0.265	1.539 ± 0.491	^∧^#	8.26E-05
	5	1.425 ± 0.418	1.103 ± 0.270	1.595 ± 0.531	^∧^#	1.34E-04
	6	1.447 ± 0.414	1.108 ± 0.284	1.623 ± 0.561	^∧^#	7.87E-05
	7	1.472 ± 0.420	1.120 ± 0.292	1.646 ± 0.568	^∧^#	6.90E-05
	8	1.500 ± 0.458	1.124 ± 0.289	1.686 ± 0.582	^∧^#	7.33E-05
	9	1.529 ± 0.463	1.135 ± 0.282	1.706 ± 0.598	^∧^#	6.30E-05
	10	1.548 ± 0.473	1.136 ± 0.301	1.730 ± 0.616	^∧^#	7.25E-05
Entropy (PWA)	1	4.539 ± 0.862	5.100 ± 0.632	5.184 ± 0.526	#	9.53E-05
	2	4.913 ± 0.745	5.385 ± 0.567	5.467 ± 0.506	#	1.03E-04
	3	4.999 ± 0.742	5.441 ± 0.621	5.543 ± 0.537	#	9.09E-05
	4	5.128 ± 0.682	5.579 ± 0.636	5.639 ± 0.539	#	7.11E-05
	5	5.253 ± 0.645	5.674 ± 0.553	5.742 ± 0.556	#	8.77E-05
	6	5.345 ± 0.634	5.776 ± 0.564	5.832 ± 0.547	#	6.55E-05
	7	5.433 ± 0.628	5.879 ± 0.595	5.887 ± 0.571	#	9.14E-05
	8	5.508 ± 0.604	5.923 ± 0.582	5.939 ± 0.582	#	7.75E-05
	9	5.567 ± 0.611	5.977 ± 0.578	5.975 ± 0.596	#	7.75E-05
	10	5.601 ± 0.604	6.013 ± 0.573	6.013 ± 0.593	#	9.49E-05
Entropy (PTT)	1	3.016 ± 1.137	3.586 ± 1.027	3.925 ± 0.980	#	1.47E-04
	2	3.324 ± 1.089	3.785 ± 0.961	4.144 ± 0.982	#	3.59E-04
	3	3.390 ± 1.093	3.803 ± 1.006	4.155 ± 0.968	#	4.12E-04
	4	3.419 ± 1.072	3.887 ± 1.015	4.206 ± 0.948	#	5.17E-04
	5	3.543 ± 1.029	3.960 ± 0.993	4.262 ± 0.964	#	4.02E-04
	6	3.579 ± 0.981	3.991 ± 0.978	4.302 ± 0.974	#	3.72E-04
	7	3.594 ± 0.976	4.031 ± 0.993	4.328 ± 0.955	#	4.18E-04
	8	3.637 ± 0.972	4.064 ± 0.984	4.370 ± 0.953	#	3.88E-04
	9	3.732 ± 0.963	4.071 ± 0.982	4.377 ± 0.948	#	4.31E-04
	10	3.746 ± 0.961	4.079 ± 0.987	4.369 ± 0.951	#	4.61E-04
Entropy (PWV)	1	3.017 ± 1.136	3.585 ± 1.022	3.922 ± 0.980	#	1.47E-04
	2	3.324 ± 1.087	3.782 ± 0.961	4.147 ± 0.980	#	3.07E-04
	3	3.387 ± 1.098	3.790 ± 1.008	4.157 ± 0.968	#	3.67E-04
	4	3.416 ± 1.075	3.886 ± 1.015	4.205 ± 0.952	#	5.34E-04
	5	3.539 ± 1.028	3.962 ± 0.989	4.262 ± 0.965	#	4.02E-04
	6	3.585 ± 0.978	3.994 ± 0.980	4.301 ± 0.967	#	3.73E-04
	7	3.594 ± 0.981	4.034 ± 0.992	4.333 ± 0.958	#	4.02E-04
	8	3.636 ± 0.976	4.062 ± 0.992	4.372 ± 0.954	#	4.33E-04
	9	3.722 ± 0.956	4.067 ± 0.986	4.370 ± 0.945	#	3.87E-04
	10	3.742 ± 0.954	4.079 ± 0.984	4.365 ± 0.953	#	4.64E-04

^*^Significantly different between MDDSI+ and MDDSI−;

∧ Significantly different between MDDSI− vs. CONT;

# *Significantly different between CONT and MDDSI+*.

Discriminative performances of all features, which were extracted using ROC curve analysis is shown in Tables 5–7. Tone values of Diastole and PWA (Table 6) had the highest AUC (Diastole 0.80–0.84 for lags 1–8 and PWA 0.80–0.81 for lags 6–10) to discriminate between MDDSI+ and MDDSI− and were better at discriminating between the MDDSI− and CONT groups (AUC 0.81 ~ 0.83 for lag 1-2). Similarly AUCs of Tone PWA and PTT were higher than other features and better at discriminating between MDDSI+ and CONT groups (Maximum AUC 0.79). Entropy-based analysis was sensitive to different aspects of the PPG recordings as shown in Table 7.

**Table 5:** Performance parameters from ROC analysis for assessing time domain features.

**Feature**		**MDDSI+ vs. MDDSI−**					**MDDSI− vs. CONT**					**CONT vs. MDDSI+**				
		**AUC**	**Th**	**Sen**	**Spec**	**Acc**	**AUC**	**Th**	**Sen**	**Spec**	**Acc**	**AUC**	**Th**	**Sen**	**Spec**	**Acc**
Systole	Mean	0.75	0.62	0.94	0.53	0.74	0.71	0.64	0.72	0.69	0.71	0.57	0.64	0.07	1.00	0.68
	SD	0.72	0.63	0.88	0.67	0.77	0.68	0.65	0.62	0.88	0.71	0.50	0.59	0.13	0.97	0.68
	RMSSD	0.69	0.62	0.94	0.60	0.77	0.79	0.63	0.76	0.81	0.78	0.65	0.63	0.13	1.00	0.70
Diastole	Mean	0.77	0.64	0.81	0.73	0.77	0.73	0.63	0.83	0.56	0.73	0.55	0.62	0.07	1.00	0.68
	SD	0.73	0.63	0.81	0.67	0.74	0.69	0.65	0.59	0.88	0.69	0.50	0.59	0.00	1.00	0.66
	RMSSD	0.72	0.64	0.88	0.67	0.77	0.81	0.63	0.79	0.88	0.82	0.61	0.61	0.13	1.00	0.70
PWA	Mean	0.75	0.63	0.94	0.53	0.74	0.72	0.63	0.83	0.56	0.73	0.56	0.63	0.07	1.00	0.68
	SD	0.70	0.63	0.88	0.60	0.74	0.68	0.65	0.59	0.88	0.69	0.52	0.59	0.00	1.00	0.66
	RMSSD	0.69	0.63	0.75	0.67	0.71	0.78	0.63	0.72	0.81	0.76	0.65	0.62	0.13	1.00	0.70
PTT	Mean	0.61	0.65	0.40	0.86	0.62	0.62	0.65	0.86	0.40	0.70	0.51	0.59	0.07	1.00	0.70
	SD	0.60	0.61	0.73	0.57	0.66	0.56	0.65	0.93	0.20	0.68	0.69	0.61	0.36	0.93	0.74
	RMSSD	0.67	0.61	0.80	0.57	0.69	0.62	0.64	1.00	0.13	0.70	0.76	0.62	0.43	1.00	0.81
PWV	Mean	0.64	0.63	0.67	0.71	0.69	0.58	0.65	0.86	0.33	0.68	0.54	0.60	0.07	1.00	0.70
	SD	0.57	0.62	0.67	0.64	0.66	0.59	0.65	0.93	0.27	0.70	0.66	0.62	0.07	1.00	0.70
	RMSSD	0.65	0.62	0.67	0.64	0.66	0.61	0.64	1.00	0.20	0.73	0.76	0.62	0.36	1.00	0.79
Age	Mean	0.65	0.63	0.80	0.57	0.69	0.71	0.66	0.90	0.53	0.77	0.79	0.61	0.57	0.97	0.84
BMI	Mean	0.69	0.63	0.93	0.50	0.72	0.60	0.65	0.79	0.53	0.70	0.76	0.59	0.43	1.00	0.81
WC	Mean	0.73	0.61	0.87	0.57	0.72	0.67	0.62	1.00	0.20	0.73	0.84	0.64	0.57	1.00	0.86

^*^AUC, Area Under ROC; Th, Threshold; Sen, Sensitivity; Spec, Specificity; Acc, Accuracy. Mean, Standard deviation (SD) and RMSSD, Root Mean Squared of Successive Difference. Underlined values indicate the significant (p < 0.05) ones.

**Table 6:** Performance parameters from ROC analysis of multilag tones.

**Feature**	**Lag, m**	**MDDSI+ vs. MDDSI−**					**MDDSI− vs. CONT**					**CONT vs. MDDSI+**				
		**AUC**	**Th**	**Sen**	**Spec**	**Acc**	**AUC**	**Th**	**Sen**	**Spec**	**Acc**	**AUC**	**Th**	**Sen**	**Spec**	**Acc**
Tone (Systole)	1	0.62	0.61	0.81	0.50	0.66	0.66	0.66	0.62	0.75	0.67	0.51	0.60	0.00	1.00	0.64
	2	0.71	0.61	0.81	0.63	0.72	0.67	0.66	0.69	0.63	0.67	0.52	0.59	0.19	0.93	0.67
	3	0.71	0.61	0.88	0.63	0.75	0.68	0.66	0.55	0.88	0.67	0.52	0.59	0.19	0.93	0.67
	4	0.71	0.61	0.88	0.63	0.75	0.69	0.66	0.66	0.75	0.69	0.52	0.59	0.19	0.93	0.67
	5	0.70	0.61	0.88	0.63	0.75	0.70	0.66	0.69	0.75	0.71	0.51	0.59	0.19	0.93	0.67
	6	0.70	0.61	0.81	0.63	0.72	0.70	0.66	0.69	0.75	0.71	0.50	0.59	0.19	0.93	0.67
	7	0.70	0.61	0.81	0.63	0.72	0.72	0.66	0.72	0.75	0.73	0.52	0.59	0.19	0.93	0.67
	8	0.69	0.61	1.00	0.44	0.72	0.71	0.66	0.66	0.81	0.71	0.53	0.59	0.19	0.93	0.67
	9	0.69	0.61	0.81	0.56	0.69	0.71	0.66	0.72	0.75	0.73	0.53	0.59	0.19	0.93	0.67
	10	0.68	0.61	0.81	0.56	0.69	0.72	0.66	0.72	0.75	0.73	0.53	0.59	0.19	0.93	0.67
Tone (Diastole)	1	0.84	0.63	0.81	0.81	0.81	0.83	0.65	0.76	0.88	0.80	0.52	0.59	0.13	0.97	0.67
	2	0.83	0.63	0.81	0.81	0.81	0.81	0.66	0.72	0.94	0.80	0.52	0.59	0.31	0.90	0.69
	3	0.82	0.63	0.81	0.81	0.81	0.77	0.65	0.76	0.81	0.78	0.55	0.60	0.25	0.93	0.69
	4	0.82	0.62	0.88	0.81	0.84	0.76	0.66	0.69	0.88	0.76	0.55	0.60	0.25	0.93	0.69
	5	0.81	0.62	0.81	0.81	0.81	0.75	0.66	0.69	0.88	0.76	0.53	0.59	0.25	0.90	0.67
	6	0.82	0.62	0.88	0.81	0.84	0.73	0.65	0.72	0.75	0.73	0.56	0.62	0.00	1.00	0.64
	7	0.82	0.62	0.88	0.81	0.84	0.72	0.66	0.62	0.88	0.71	0.56	0.62	0.00	1.00	0.64
	8	0.81	0.62	0.75	0.81	0.78	0.72	0.66	0.62	0.88	0.71	0.56	0.62	0.00	1.00	0.64
	9	0.80	0.62	0.75	0.81	0.78	0.71	0.65	0.69	0.75	0.71	0.57	0.62	0.00	1.00	0.64
	10	0.79	0.62	0.75	0.81	0.78	0.69	0.66	0.66	0.75	0.69	0.57	0.62	0.00	1.00	0.64
Tone (PWA)	1	0.75	0.61	0.94	0.60	0.77	0.54	0.65	1.00	0.06	0.67	0.70	0.61	0.27	1.00	0.75
	2	0.77	0.61	0.88	0.60	0.74	0.55	0.65	0.93	0.13	0.64	0.70	0.61	0.27	1.00	0.75
	3	0.78	0.61	0.88	0.67	0.77	0.58	0.65	0.97	0.13	0.67	0.71	0.62	0.20	1.00	0.73
	4	0.79	0.61	0.88	0.73	0.81	0.59	0.64	1.00	0.13	0.69	0.70	0.62	0.27	1.00	0.75
	5	0.78	0.60	0.94	0.73	0.84	0.62	0.63	1.00	0.06	0.67	0.70	0.62	0.27	1.00	0.75
	6	0.80	0.61	0.88	0.73	0.81	0.63	0.62	0.97	0.13	0.67	0.71	0.62	0.27	1.00	0.75
	7	0.80	0.60	0.94	0.73	0.84	0.66	0.66	0.72	0.63	0.69	0.71	0.62	0.27	1.00	0.75
	8	0.80	0.61	0.94	0.73	0.84	0.66	0.66	0.76	0.63	0.71	0.70	0.61	0.47	0.90	0.75
	9	0.80	0.61	0.94	0.73	0.84	0.69	0.66	0.76	0.63	0.71	0.68	0.61	0.47	0.90	0.75
	10	0.81	0.61	0.94	0.73	0.84	0.70	0.67	0.55	0.94	0.69	0.68	0.61	0.33	0.97	0.75
Tone (PTT)	1	0.70	0.61	0.80	0.64	0.72	0.60	0.64	1.00	0.20	0.73	0.79	0.60	0.57	1.00	0.86
	2	0.66	0.61	0.73	0.64	0.69	0.62	0.64	0.90	0.33	0.70	0.77	0.61	0.50	0.97	0.81
	3	0.68	0.61	0.80	0.64	0.72	0.63	0.64	0.93	0.33	0.73	0.77	0.61	0.64	0.93	0.84
	4	0.67	0.61	0.73	0.64	0.69	0.61	0.64	0.90	0.40	0.73	0.77	0.61	0.50	0.97	0.81
	5	0.63	0.62	0.60	0.71	0.66	0.60	0.64	0.93	0.33	0.73	0.76	0.61	0.57	0.93	0.81
	6	0.62	0.63	0.60	0.71	0.66	0.61	0.64	0.86	0.40	0.70	0.76	0.61	0.64	0.90	0.81
	7	0.61	0.63	0.60	0.71	0.66	0.61	0.64	0.93	0.33	0.73	0.76	0.61	0.64	0.90	0.81
	8	0.62	0.63	0.60	0.71	0.66	0.60	0.64	0.90	0.33	0.70	0.77	0.61	0.57	0.90	0.79
	9	0.60	0.62	0.60	0.71	0.66	0.60	0.64	0.90	0.33	0.70	0.75	0.61	0.57	0.90	0.79
	10	0.58	0.62	0.60	0.71	0.66	0.59	0.64	0.93	0.27	0.70	0.74	0.61	0.50	0.90	0.77
Tone (PWV)	1	0.60	0.62	0.60	0.71	0.66	0.61	0.64	0.86	0.33	0.68	0.67	0.60	0.36	0.86	0.70
	2	0.60	0.62	0.67	0.57	0.62	0.62	0.64	1.00	0.07	0.68	0.66	0.61	0.00	1.00	0.67
	3	0.57	0.62	0.87	0.36	0.62	0.63	0.64	1.00	0.07	0.68	0.65	0.61	0.00	1.00	0.67
	4	0.54	0.63	0.40	0.79	0.59	0.63	0.64	1.00	0.07	0.68	0.65	0.61	0.00	1.00	0.67
	5	0.53	0.63	0.40	0.79	0.59	0.61	0.64	1.00	0.07	0.68	0.64	0.61	0.00	1.00	0.67
	6	0.54	0.62	0.47	0.79	0.62	0.60	0.64	1.00	0.07	0.68	0.62	0.61	0.00	1.00	0.67
	7	0.51	0.62	0.40	0.79	0.59	0.61	0.64	1.00	0.07	0.68	0.63	0.61	0.00	1.00	0.67
	8	0.53	0.63	0.33	0.86	0.59	0.60	0.64	1.00	0.07	0.68	0.62	0.61	0.00	1.00	0.67
	9	0.50	0.62	0.40	0.86	0.62	0.60	0.64	1.00	0.07	0.68	0.62	0.60	0.86	0.62	0.70
	10	0.51	0.63	0.40	0.86	0.62	0.59	0.64	1.00	0.07	0.68	0.60	0.61	0.00	1.00	0.67

^*^AUC, Area Under ROC; Th, Threshold; Sen, Sensitivity; Spec, Specificity; Acc, Accuracy. Single underlined values–AUC ≥ 0.70 and double underlined values–AUC ≥ 0.80.

**Table 7:** Performance parameters from ROC analysis of multilag entropy features.

**Feature**	**Lag, m**	**MDDSI+ vs. MDDSI−**					**MDDSI− vs. CONT**					**CONT vs. MDDSI+**				
		**AUC**	**Th**	**Sen**	**Spec**	**Acc**	**AUC**	**Th**	**Sen**	**Spec**	**Acc**	**AUC**	**Th**	**Sen**	**Spec**	**Acc**
Entropy (Systole)	1	0.71	0.63	0.88	0.63	0.75	0.75	0.64	0.66	0.94	0.76	0.60	0.62	0.06	1.00	0.67
	2	0.73	0.64	0.88	0.63	0.75	0.78	0.63	0.69	0.94	0.78	0.60	0.61	0.06	1.00	0.67
	3	0.77	0.64	0.94	0.69	0.81	0.80	0.63	0.76	0.94	0.82	0.61	0.61	0.00	1.00	0.64
	4	0.73	0.64	0.94	0.69	0.81	0.81	0.63	0.79	0.94	0.84	0.63	0.62	0.06	1.00	0.67
	5	0.72	0.64	0.94	0.69	0.81	0.79	0.63	0.79	0.94	0.84	0.59	0.61	0.06	1.00	0.67
	6	0.76	0.64	0.94	0.63	0.78	0.83	0.63	0.76	0.94	0.82	0.57	0.61	0.06	1.00	0.67
	7	0.73	0.64	0.94	0.63	0.78	0.80	0.63	0.79	0.88	0.82	0.57	0.61	0.06	1.00	0.67
	8	0.72	0.64	0.94	0.56	0.75	0.81	0.63	0.79	0.94	0.84	0.57	0.61	0.06	1.00	0.67
	9	0.71	0.64	0.88	0.63	0.75	0.81	0.63	0.79	0.94	0.84	0.58	0.61	0.06	1.00	0.67
	10	0.74	0.65	0.81	0.69	0.75	0.82	0.63	0.79	0.88	0.82	0.57	0.61	0.06	1.00	0.67
Entropy (Diastole)	1	0.78	0.64	0.94	0.63	0.78	0.85	0.63	0.76	0.94	0.82	0.61	0.61	0.13	0.97	0.67
	2	0.73	0.64	0.88	0.69	0.78	0.80	0.63	0.76	0.88	0.80	0.60	0.61	0.13	1.00	0.69
	3	0.84	0.64	0.88	0.81	0.84	0.83	0.63	0.76	0.94	0.82	0.59	0.61	0.00	1.00	0.64
	4	0.78	0.64	0.94	0.69	0.81	0.83	0.63	0.76	0.94	0.82	0.61	0.61	0.19	0.97	0.69
	5	0.79	0.64	0.81	0.75	0.78	0.84	0.62	0.79	0.88	0.82	0.60	0.61	0.06	1.00	0.67
	6	0.80	0.64	0.94	0.75	0.84	0.82	0.63	0.76	0.94	0.82	0.58	0.61	0.06	1.00	0.67
	7	0.80	0.64	0.94	0.75	0.84	0.82	0.63	0.79	0.94	0.84	0.59	0.61	0.19	0.97	0.69
	8	0.79	0.64	0.94	0.75	0.84	0.82	0.63	0.79	0.94	0.84	0.60	0.61	0.06	1.00	0.67
	9	0.82	0.64	0.94	0.81	0.88	0.82	0.63	0.79	0.94	0.84	0.59	0.61	0.13	1.00	0.69
	10	0.81	0.65	0.94	0.81	0.88	0.81	0.63	0.79	0.94	0.84	0.57	0.61	0.00	1.00	0.64
Entropy (PWA)	1	0.69	0.64	0.69	0.67	0.68	0.54	0.65	0.93	0.25	0.69	0.72	0.63	0.53	0.97	0.82
	2	0.68	0.64	0.69	0.67	0.68	0.54	0.64	0.97	0.19	0.69	0.71	0.63	0.47	0.93	0.77
	3	0.66	0.62	0.75	0.60	0.68	0.55	0.65	0.90	0.25	0.67	0.72	0.61	0.53	0.90	0.77
	4	0.68	0.63	0.75	0.67	0.71	0.53	0.64	1.00	0.06	0.67	0.72	0.62	0.47	0.90	0.75
	5	0.69	0.60	0.94	0.40	0.68	0.54	0.65	0.86	0.25	0.64	0.71	0.62	0.47	0.90	0.75
	6	0.71	0.62	0.69	0.67	0.68	0.52	0.65	0.90	0.19	0.64	0.71	0.61	0.53	0.90	0.77
	7	0.72	0.62	0.81	0.60	0.71	0.50	0.66	0.90	0.19	0.64	0.71	0.61	0.47	0.90	0.75
	8	0.70	0.63	0.56	0.80	0.68	0.51	0.65	0.90	0.19	0.64	0.69	0.61	0.40	0.90	0.73
	9	0.69	0.62	0.69	0.67	0.68	0.50	0.66	0.86	0.25	0.64	0.67	0.64	0.20	0.97	0.70
	10	0.69	0.62	0.63	0.73	0.68	0.52	0.66	0.86	0.25	0.64	0.68	0.61	0.40	0.90	0.73
Entropy (PTT)	1	0.66	0.64	0.60	0.71	0.66	0.58	0.64	0.90	0.33	0.70	0.73	0.64	0.36	1.00	0.79
	2	0.64	0.62	0.73	0.64	0.69	0.60	0.63	1.00	0.13	0.70	0.72	0.63	0.29	1.00	0.77
	3	0.60	0.61	0.80	0.50	0.66	0.60	0.63	1.00	0.20	0.73	0.71	0.63	0.29	1.00	0.77
	4	0.65	0.61	0.80	0.50	0.66	0.60	0.64	0.90	0.33	0.70	0.73	0.62	0.50	0.93	0.79
	5	0.63	0.61	0.80	0.50	0.66	0.60	0.64	0.90	0.33	0.70	0.69	0.62	0.50	0.90	0.77
	6	0.63	0.61	0.80	0.50	0.66	0.60	0.63	0.97	0.20	0.70	0.71	0.62	0.50	0.90	0.77
	7	0.63	0.64	0.53	0.71	0.62	0.60	0.63	1.00	0.20	0.73	0.73	0.63	0.29	1.00	0.77
	8	0.62	0.64	0.53	0.71	0.62	0.60	0.63	1.00	0.13	0.70	0.71	0.63	0.29	1.00	0.77
	9	0.59	0.62	0.73	0.50	0.62	0.60	0.64	0.97	0.20	0.70	0.70	0.62	0.43	0.90	0.74
	10	0.59	0.62	0.73	0.50	0.62	0.60	0.64	1.00	0.20	0.73	0.69	0.63	0.21	1.00	0.74
Entropy (PWV)	1	0.66	0.64	0.60	0.71	0.66	0.58	0.64	0.90	0.33	0.70	0.74	0.63	0.43	1.00	0.81
	2	0.64	0.62	0.73	0.64	0.69	0.60	0.63	1.00	0.13	0.70	0.72	0.63	0.29	1.00	0.77
	3	0.60	0.61	0.80	0.50	0.66	0.60	0.63	1.00	0.20	0.73	0.71	0.63	0.29	1.00	0.77
	4	0.64	0.61	0.80	0.50	0.66	0.60	0.64	0.90	0.33	0.70	0.73	0.62	0.50	0.93	0.79
	5	0.62	0.61	0.80	0.50	0.66	0.60	0.64	0.90	0.33	0.70	0.70	0.62	0.50	0.90	0.77
	6	0.63	0.61	0.80	0.50	0.66	0.60	0.64	0.97	0.20	0.70	0.71	0.62	0.50	0.90	0.77
	7	0.64	0.64	0.53	0.71	0.62	0.60	0.63	1.00	0.20	0.73	0.72	0.63	0.29	1.00	0.77
	8	0.63	0.64	0.53	0.71	0.62	0.60	0.63	1.00	0.13	0.70	0.72	0.63	0.29	1.00	0.77
	9	0.59	0.62	0.73	0.50	0.62	0.60	0.64	0.97	0.20	0.70	0.70	0.62	0.43	0.90	0.74
	10	0.59	0.62	0.73	0.50	0.62	0.60	0.64	1.00	0.20	0.73	0.69	0.63	0.21	1.00	0.74

^*^AUC, Area Under ROC; Th, Threshold; Sen, Sensitivity; Spec, Specificity; Acc-Accuracy. Single underlined values mean AUC ≥ 0.70 and double underlined values mean AUC ≥ 0.80.

**Figure 4:**
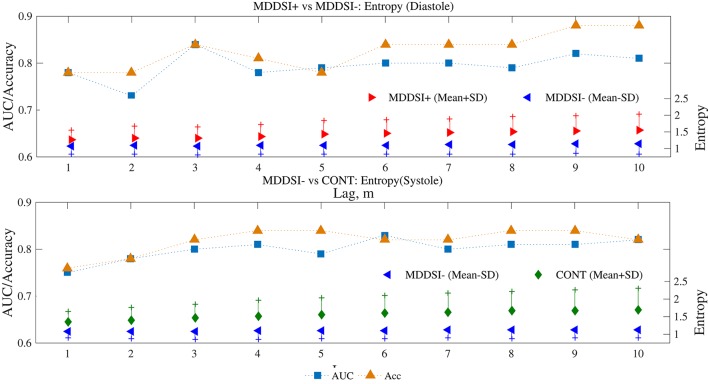
Visualization of example performances (AUC; Area under ROC curve and Acc; Accuracy) assessed by ROC analysis for three distinct combination of groups, i.e., MDDSI+ vs. MDDSI−, MDDSI− vs. CONT and CONT vs. MDDSI+ at several lags (lag, m = [1 2 3 4 5 6 7 8 9 10]).

Entropies of Systole between lags 3–10 and Diastoles for all lags discriminated (Entropy Systoles: AUC = 0.79 ~ 0.82 for lags 3 ~ 10; Entropy Diastoles AUC = 0.80 ~ 0.85 for lags 1 ~ 10) between MDDSI− and CONT groups (*p* < 0.01). As for discrimination between MDDSI+ and MDDSI− groups, Entropy values of Diastole at lags 3, 6,7,9,10 were still acceptable (AUC > 0.80) but lower than the tone results (Figure 4).

**Figure 5:**
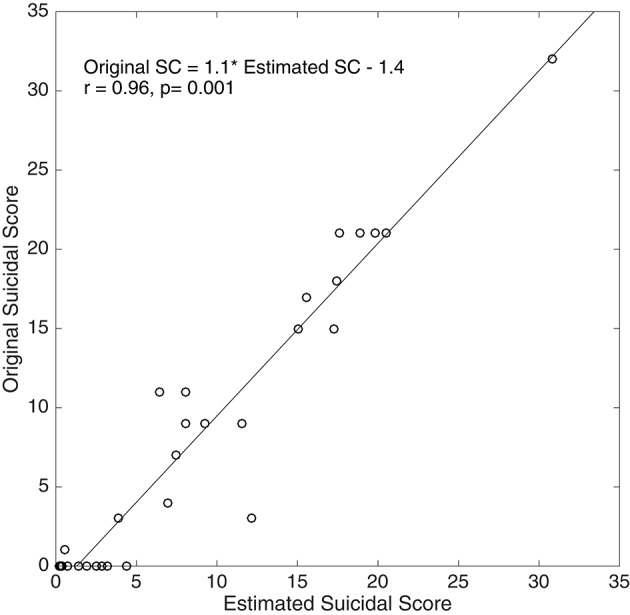
Correlation between Estimated Suicidal Score (SC) and Original SC for 30 MDDSI+/SI− subjects.

Multiple logistic regression models and classification performances of the three models for combining diastolic T-E features with and without anthropometric features for Model 1 (MDDSI+/MDDSI−) and Model 2 (MDDSI−/CONT), and PTT T-E features for Model 3 (MDDSI+/CONT) are shown in Tables 8A,B respectively. Model 1 (Table 8A), built on diastolic T-E features was shown to be the best indicator (accuracy = 93.33%; PPV = 100% for MDDSI+ and NPV = 87.5% for MDDSI−) in classifying MDDSI+/MDDSI- for all lags apart from lag 9. The second model (Table 8A), built on diastolic T-E features at lag 2,6, and 8 was able to accurately classify MDDSI−/CONT groups with accuracy of 93.18% (PPV = 87.5% for MDDSI− and NPV = 96.43% for CONT). The third model (Table 8A), which was built on PTT multilag T-E features classified MDDSI+/CONT groups at most lags with an acceptable accuracy for clinical risk assessment accuracy of 92.85% (PPV = 85.71% for MDDSI+ and NPV = 96.43% for CONT). Improvements were obtained when anthropometric features were added as the overall performance of all three models consequently improved (Table 8B). Applying a continuous model (in Table 8C) showed that BMI, WC, Tone and Entropy were significantly associated with suicidal scores. Figure 5 showed the correlation (*r* = 0.96; *p* = 0.001) between the estimated and original suicidal scores.

**Table 8A:** Multiple stepwise logistic regression models for predicting MDDSI+/− from multilag tone entropy features.

	**Estimate**	**SE**	***t*-stats**	***p*-value**	**Stats**	**Performance**
**MODEL 1_DIASTOLE (MDDSI+/MDDSI−)**						
Intercept	7.43	11.72	0.63	0.53	*F* = 2.8 *p* = 0.03, *R*^2^ = 0.40	AUC = 0.96 Acc = 93.33% PPV = 100% NPV = 87.5%
Entropy 2	−6.39	1.92	−3.33	3.69E-03		
Tone 2	−25.98	12.07	−2.15	0.05		
Entropy 3	5.40	2.10	2.57	0.02		
Entropy 4	6.14	2.69	2.28	0.03		
Tone 4	37.73	14.67	2.57	0.02		
Tone 5	−68.21	22.02	−3.10	0.01		
Tone 6	108.51	30.17	3.60	2.06E-03		
Entropy 8	−9.20	3.76	−2.44	0.03		
Tone 8	−78.96	23.18	−3.41	3.14E-03		
Entropy 10	6.12	2.54	2.40	0.03		
Tone 10	22.32	8.83	2.53	0.02		
**MODEL 2_DIASTOLE (MDDSI−/CONT)**						
Intercept	−16.92	5.17	−3.27	2.23E-03	*F* = 7.6 *p* = 1.26E-04, *R*^2^ = 0.50	AUC = 0.96 Acc = 93.18% PPV = 87.5% NPV = 96.43%
Tone 2	12.61	3.72	3.3873	1.62E-03		
Entropy 2	2.02	1.01	2.01	0.05		
Tone 6	−3.89	1.21	−3.20	2.71E-03		
Entropy 8	−1.96	0.89	−2.21	0.03		
**MODEL 3_PTT (MDDSI+/CONT)**						
Intercept	−0.03	0.40	−0.09	0.93	*F* = 4.71 *p* = 3.47E-04, *R*^2^ = 0.38	AUC = 0.97 Acc = 92.85% PPV = 85.71% NPV = 96.43%
Entropy 1	−1.12	0.27	−4.23	2.02E-04		
Entropy 3	2.77	0.69	4.04	3.44E-04		
Tone 3	0.38	0.16	2.42	0.02		
Entropy 4	−2.63	0.70	−3.76	7.30E-04		
Tone 4	−0.46	0.22	−2.10	0.04		
Tone 5	−0.26	0.14	−1.84	0.08		
Tone 6	0.70	0.21	3.39	2.00E-03		
Tone 7	−0.66	0.18	−3.72	8.28E-04		
Tone 8	0.72	0.16	4.48	1.00E-04		
Entropy 10	0.98	0.35	2.84	0.01		
Tone 10	−0.38	0.09	−4.37	1.38E-04		

*AUC, Area Under ROC; Acc, Accuracy; PPV, Positive Predictive Value; NPV, Negative Predictive Value*.

**Table 8B:** Multiple stepwise logistic regression models for predicting MDDSI+/−.

	**Estimate**	**SE**	***t*-stats**	***p*-value**	***F*-stats**	**Performance**
**MODEL 1_DIASTOLE (MDDSI+/MDDSI−)**						
Intercept	−23.15	12.54	−1.85	0.08	*F* = 3.99 *p* = 0.01 *R*^2^ = 0.11	AUC = 0.99 Acc = 96.67% PPV = 100% NPV = 93.75%
WC	0.01	0.01	2.50	2.35E-02		
Tone1	39.14	12.00	3.26	4.91E-03		
Entropy2	−6.29	1.82	−3.45	3.30E-03		
Tone2	−55.26	14.89	−3.71	1.89E-03		
Entropy3	8.53	2.13	4.00	1.03E-03		
Entropy4	7.25	2.86	2.54	0.02		
Tone4	38.06	11.29	3.37	3.88E-03		
Tone5	−67.73	17.28	−3.92	1.22E-03		
Tone6	117.38	25.48	4.61	2.91E-04		
Entropy7	−13.89	3.83	−3.62	2.29E-03		
Tone 9	−88.62	21.47	−4.13	7.90E-04		
Entropy10	7.14	2.09	3.41	3.57E-03		
Tone10	26.81	8.31	3.23	0.01		
**MODEL 2_DIASTOLE (MDDSI**−**/CONT)**						
Intercept	−18.09	4.48	−4.04	2.71E-04	*F* = 8.08 *p* = 6.87E-06 *R*^2^ = 0.11	AUC = 0.98 Acc = 93.18% PPV = 87.5% NPV = 96.43%
Age	0.02	0.01	2.53	1.60E-02		
BMI	−0.07	0.03	−2.54	1.55E-02		
WC	0.03	0.01	3.34	1.96E-03		
Tone2	12.73	3.23	3.94	3.54E-04		
Entropy5	2.53	0.91	2.78	8.60E-03		
Tone6	−4.02	1.05	−3.83	4.95E-04		
Entropy8	−2.42	0.80	−3.01	4.71E-03		
**MODEL 3_PTT (MDDSI+/CONT)**						
Intercept	−1.86	0.44	−4.26	1.70E-04	*F* = 13.1 *p* = 1.93E-08 *R*^2^ = 0.06	AUC = 0.99 Acc = 97.62% PPV = 92.86% NPV = 100%
Age	0.02	4.79E-03	4.20	2.00E-04		
WC	0.01	3.00E-03	4.35	1.29E-04		
Entropy1	−0.88	0.17	−5.08	1.58E-05		
Entropy3	0.70	0.31	2.22	3.33E-02		
Entropy7	−0.97	0.52	−1.87	0.07		
Tone7	−0.16	0.06	−2.77	0.01		
Tone8	0.47	0.12	3.84	5.46E-04		
Tone9	−0.31	0.09	−3.29	2.47E-03		
Entropy10	1.18	0.39	3.01	5.08E-03		

*AUC, Area Under ROC; Acc, Accuracy; PPV, Positive Predictive Value; NPV, Negative Predictive Value. BMI, Body Mass Index; WC, Waist Circumference*.

**Table 8C:** Multiple stepwise regression models for predicting suicidal scores (SC) of MDDSI+/− patients as a continuous measure.

	**Estimate**	**SE**	***t*-stats**	***p*-value**	***F*-stats**
**MODEL 1_DIASTOLE (MDDSI+/MDDSI−)**					
Intercept	−102.91	14.90	−6.91	1.64E-05	*F* = 7.29 *p* = 0.006 *R*^2^ = 0.91 *R*^2^ = 0.78 (Adjusted)
BMI	−0.24	0.08	−2.81	1.59E-02	
WC	0.50	0.10	4.79	4.41E-04	
Tone2	−1.64E04	3,750.70	−4.39	8.81E-04	
Entropy3	276.50	47.52	5.82	8.23E-05	
Tone3	0.78E04	3,117.10	2.51	2.75E-02	
Entropy4	142.91	37.92	3.77	2.68E-03	
Tone4	0.41E04	1,992.20	2.07	6.12E-02	
Tone5	−1.44E04	2,422.10	−5.98	6.38E-05	
Entropy6	−108.43	47.07	−2.30	3.99E-02	
Tone6	2.83E04	3,974.10	7.12	1.21E-05	
Entropy7	−342.43	67.69	−5.06	2.80E-04	
Tone7	−0.53E04	2,948.60	−1.83	9.28E-02	
Tone9	−1.89E04	2,792.60	−6.79	1.93E-05	
Entropy10	241.70	44.21	5.47	1.44E-04	
Tone10	7,416.3	1,260.2	5.8849	7.42E-05	

*Estimated SC = Intercept+E1 × BMI+E2 × WC+E3 × Tn2+E4 × Ent3+E5 × Tn3+E5 × Ent4+E6 × Tn4+E7 × Tn5+E8 × Ent6+E9 × Tn6+E10 × Ent7+E11 × Tn7+E12 × Tn9+E13 × Ent10+E14 × Tn10*.

Correlations between the suicidal score and individual features are summarized in Table 9. RMSSD and Entropy values of Systole (except lags 1 and 4) were significantly correlated with suicidal score (*p* < 0.05). RMSSD and Entropy values of Diastoles at all lags were also correlated with suicidal score (*p* < 0.05). The highest correlation coefficient was found to be 0.49 for Entropy of Diastole at lag 3.

**Table 9:** Correlation coefficients (*r*) of time domain and multi-lag T-E features with suicidal scores.

**Lag, m**	**Feature**	**Systole**		**Diastole**		**PWA**		**PTT**		**PWV**	
		***r***	***p*-value**	***r***	***p*-value**	***r***	***p*-value**	***r***	***p*-value**	***r***	***p*-value**
1	Entropy	0.29	0.13	**0.4**	**0.03**	0.07	0.71	0.12	0.54	0.12	0.53
	Tone	−0.07	0.7	−0.05	0.79	−0.06	0.75	−0.04	0.83	−0.03	0.88
2	Entropy	**0.43**	**0.02**	**0.46**	**0.01**	0.1	0.63	0.15	0.43	0.16	0.43
	Tone	−0.02	0.93	−0.02	0.9	−0.06	0.74	−0.09	0.64	−0.05	0.8
3	Entropy	**0.42**	**0.03**	**0.49**	**0.01**	0.09	0.66	0.15	0.45	0.15	0.44
	Tone	0	1	−0.02	0.92	0.01	0.94	−0.08	0.7	−0.05	0.79
4	Entropy	0.35	0.07	**0.37**	**0.05**	0.03	0.87	0.09	0.65	0.09	0.64
	Tone	0.02	0.91	−0.01	0.94	0	0.99	−0.05	0.79	0.02	0.92
5	Entropy	**0.4**	**0.03**	**0.42**	**0.03**	0	0.99	0.11	0.58	0.11	0.59
	Tone	0.02	0.92	0	0.99	0.02	0.92	−0.08	0.68	0.01	0.97
6	Entropy	**0.39**	**0.04**	**0.47**	**0.01**	0.01	0.97	0.11	0.57	0.11	0.57
	Tone	0.01	0.95	0.01	0.96	0.03	0.89	−0.05	0.8	0.04	0.84
7	Entropy	**0.4**	**0.03**	**0.43**	**0.02**	−0.01	0.94	0.09	0.66	0.08	0.67
	Tone	0.01	0.97	0.01	0.96	0.05	0.82	−0.01	0.95	0.06	0.75
8	Entropy	**0.39**	**0.04**	**0.4**	**0.03**	−0.02	0.93	0.09	0.67	0.08	0.67
	Tone	0.01	0.96	0.01	0.94	0.08	0.69	−0.02	0.92	0.05	0.78
9	Entropy	**0.39**	**0.04**	**0.45**	**0.02**	−0.01	0.97	0.11	0.58	0.11	0.56
	Tone	0.01	0.96	0.01	0.95	0.06	0.75	−0.05	0.8	0.06	0.75
10	Entropy	**0.39**	**0.04**	**0.45**	**0.02**	−0.02	0.92	0.12	0.55	0.12	0.56
	Tone	0.01	0.94	0.02	0.93	0.08	0.67	−0.08	0.67	0.08	0.7
	Mean	0.19	0.34	−0.28	0.14	0.23	0.23	−0.03	0.88	−0.23	0.24
	SD	0.21	0.29	0.29	0.14	0.24	0.22	0.08	0.70	−0.01	0.95
	RMSSD	**0.41**	**0.03**	**0.44**	**0.02**	0.48	0.01	0.07	0.74	0.08	0.69

*Bold-underlined values indicate the significant (p < 0.05) ones. PWA, Pulse Wave Amplitude; PTT, Pulse Transit Time; PWV, Pulse Wave Velocity*.

## Discussion

This study examined whether or not photo-plethysmography could be used for monitoring MDD patients to identify suicidal risk. The main finding was that suicidal ideation in MDD patients correlated with arterial pulse wave features. Univariate analyses showed that Tone (Diastole) and Entropy (Diastole) values at all lags were the best discriminator (AUC >0.8) between MDDSI+/MDDSI− and between MDDSI−/CONT groups (Figure 3). Multivariate logistic regression modeling showed that suicidal risk was significantly associated with Tone and Entropy values at lags more than 1, which justified the importance of using T-E features at higher lags. Moreover, as indicated by the results of logistic regression analysis, the feature set Tone and Entropy of Diastole demonstrated statistically significant predictive power for assessing MDD patients at risk of suicidal ideation. The identified predictors Tone and Entropy were shown to classify MDDSI-patients (without suicidal ideation) from the control group, which could eventually also help mental health care providers screen the risk of MDD without suicidal ideation in an efficient, convenient, and non-invasive manner. However, the time domain RMSSD parameter, which is analogous to short lags and an indicator of parasympathetic function, did not discriminate very well between the groups, suggesting that depression and suicidal ideation may be more sympathetically driven as reported by previous studies (Barton et al., 2007; Licht et al., 2012). Considering the importance of multilag analysis, higher lags mean slower oscillations in PPG features and are more likely to reflect sympathetic function. The association of higher lags for Tone and Entropy features might therefore be due to the activation and deactivation of the sympathetic nervous system in MDD suicidal ideation subjects.

The Systole point in the PPG signal indicates the end systolic volume at peak ventricular ejection, which was measured from the maximum arterial pressure, which occurs during contraction of the left ventricle. The peripheral resistance in the blood vessel influences the systolic value. The tone of systole measured in the current experiments represents the balance (or control) between increasing and decreasing peripheral resistance (related to sympathetic activation and deactivation); whereas entropy of systole represents the overall changes in peripheral resistance. The diastole point refers to the end diastolic volume, indicated by the minimum arterial pressure during relaxation and dilatation of the ventricles of the heart, when the ventricles fill with blood. During diastole the arterial walls are still stretched by the remaining blood, and blood continues to be driven into arterioles. Specifically reduction of arterial compliance (increased arterial stiffness) leads to a higher systolic (SBP) and a lower diastolic blood pressure (DBP) (Hilden, 1991). A highly compliant aorta (i.e., less stiff, normal aorta) has a smaller pulse pressure for a given stroke volume into the aorta than a stiff, low compliant aorta. For a given stroke volume, compliance determines pulse pressure. PWA of PPG signal measures the difference of vascular blood volume from the systolic to diastolic peaks (Miyasaka et al., 1978). Increasing arterial stiffness also leads to increased systolic blood pressure and a widening pulse pressure reflected as a higher PWA (Steppan et al., 2011) and results in an increase in Pulse Wave Velocity (Laurent and Boutouyrie, 2007). The amplification of pulse pressure can be quantified by measuring the PWA (surrogate of pulse pressure). Tone and entropy of PWA (given PWA is proportional to pulse pressure) represents balance (control) and overall activity of increasing and decreasing pulse pressure. The current results can be interpreted as supporting previous studies linking absolute values of PPG parameters with arterial stiffness as increased PWA values in the MDDSI+ group may have been caused by arterial stiffness, which in turn reflects greater variability in sympathetic activation. However, there are no studies on Tone-Entropy values associated with PPG parameters and arterial stiffness. These results support earlier findings (Broadley et al., 2002; Sherwood et al., 2005) that compliance in blood vessels and associated blood flow characteristics play a part in the extended symptomatology of MDD. We speculate that Tone and Entropy values of pulse wave parameters may be associated with vascular neuro-endocrine control mechanisms (balance between activation and deactivation of sympathetic nervous system, renin-angiotensin system, vasopressin and atrial natriuretic peptide). Our entropy values however may also be associated with sympathetic activation as increased PWA values are associated with sympathetic activation, which is supported by previous studies (Broadley et al., 2002; Sherwood et al., 2005). In addition loss of blood vessel compliance and the associated arterial stiffness have also been shown to be part of the extended symptomatology of MDD (Mannie et al., 2013). These previous findings suggest that there is a close relationship between PPG measures and hence our Tone-Entropy results, which show significant differences between groups may be a novel interpretation of pulse wave variability and sensitive to MDD and suicidality.

Physiological factors such as endothelial dysfunction have been shown to lead to the development and progression of coronary heart disease (CHD) and changes in cognitive function associated with suicidal ideation in MDD patients (Bush et al., 2001; Wulsin and Singal, 2003). The relationship between sympathetic activity of autonomic nervous system (ANS) and the main factors affecting arterial distensibility or stiffness is complex. The sympathetic nervous system is considered one of the major elements involved in the regulation of mean arterial pressure, affecting heart rate, left ventricular contractility, and systemic vascular resistance (Charkoudian and Rabbitts, 2009). How the magnitude of PWA is related to endothelial dysfunction is still unknown and remains to be investigated. It is however possible that the higher PWA could be related to changes in smooth muscle cell function or other mechanical properties of the resistance of vessels. Future research endeavors should evaluate the mechanism through which depressive symptoms with suicidal ideation influence the vascular endothelial function by investigating the biochemical markers of blood vessels and linking them with PPG based pulse wave variability features. How tone and entropy of the PPG features are directly associated with neurobiology and biochemistry of suicidal ideation in MDD patients requires further research. However, understanding the pathophysiological mechanism underlying the association of these features with depression and suicidal ideation could provide deeper insight into biological links with PPG features. Previous research have shown that vascular disease and related brain pathologies (e.g., stroke, silent brain infarction, and subclinical brain injury) are associated with cognitive decline, dementia and depression (Seldenrijk et al., 2011). Arterial stiffness is one of the earliest manifestations of adverse structural and functional changes within the arterial wall, due to inflammation, oxidative stress, atherosclerosis, and aging (Anderson, 2006). Ventricles ejecting into a stiffer arterial system must generate higher end-systolic pressures for the same net stroke volume. Increased arterial stiffness was reported to be associated with increased catecholamine level during noradrenalin infusion in a small group of patients (Wittrock et al., 2009), which caused sympathetic activation. On the other hand, decreased arterial stiffness was correlated with acute and chronic mental stress (Gorelick et al., 2011), depression and anxiety (Seldenrijk et al., 2011). Oulis and co-researchers documented reversal of arterial stiffness in a small group of severely depressed women, after a 6-week antidepressant therapy (Selective serotonin re-uptake inhibitors, Serotonin–norepinephrine reuptake inhibitors or a combination thereof in adequate dosages; Oulis et al., 2010), suggesting a pathophysiological connection. Postmortem tissue analysis of depressed suicide victims showed alterations in the density and affinity of β and α2-adrenergic receptors, which play an important role in vasodynamics and are associated with the sympathetic branch of the ANS. However, the biochemical abnormalities underlying the predisposition to and the pathogenesis of suicidal ideation in MDD and their arterial vasodynamics remain to be clearly established.

Results of the multiple logistic regression models in our study have clearly indicated a significant association between WC and age among the MDD patients with suicidal ideation; in comparison with MDD patients without suicidal ideation and the control group respectively. BMI and abdominal obesity (measured by WC) have recently been associated with arterial stiffness in different populations in Europe and the US (Budimir et al., 2012; Urbina et al., 2012) and suicidal ideation among US Adult Women (Zhao et al., 2011). Arterial walls stiffen with age and endothelial dysfunction is characteristic of arterial aging (Lee and Oh, 2010). As shown in Table 8B, improvements in classification performance by adding multi-lag T-E features with anthropometric features (Age and WC) indicate the importance of using combined feature subsets to stratify the risk of suicidal ideation. Our results strongly support the previous findings of associations between suicidal ideation and WC. Other studies suggest that a long-term increased production of stress hormones from hypothalamic-pituitary-adrenal (HPA) axis is involved in depression with suicidal risk, which contributes to body fat accumulation (Bjorntorp, 2001; Zhao et al., 2011).

In conclusion the warning signs of suicidal risk are largely subjective and could be missed by individuals and in some cases by experts leading to catastrophic outcomes. Suicide is not always a consequence of major depression as reported by Pompili (2010) that suicide might be better understood as a phenomenon centered in the individual, particularly variables predicting suicidal behavior should consider individual personality in addition to other existing measures (Pompili, 2010). Therefore, our findings are significant as we show that PPG associated features are related with suicidal ideation among our patient cohort and provides a basis for effective treatment by simply monitoring tone-entropy values of the PPG features. Determining tone and entropy from PPG is a simple and non-invasive test that can be easily incorporated into clinical settings. However, more research on multivariate analyses of T-E features of PPG signals and a larger sample size is required to validate the clinical utility of TE analysis in future research.

## Ethics statement

This study was carried out in accordance with the recommendations of Al Ain District Ethics Committee with written informed consent from all subjects. All subjects gave written informed consent in accordance with the Declaration of Helsinki. The protocol (14/28) was approved by the Al Ain District Ethics Committee and United Arab Emirates University Ethics Committee.

## Author contributions

AK conceived, derived and implemented the tone entropy analysis, generated experimental results data and drafted the Manuscript. SS generated MATLAB codes and the plots for multi-lag tone entropy analysis. VL and YA carried out the experiments at American Center for Psychiatry and Neurology in Abu Dhabi. KA, RM, NC, and HJ contributed to the manuscript, participated in the discussion and interpretation of the results. All authors read and approved the final manuscript.

### Conflict of interest statement

The authors declare that the research was conducted in the absence of any commercial or financial relationships that could be construed as a potential conflict of interest.
